# A Comprehensive Look at In Vitro Angiogenesis Image Analysis Software

**DOI:** 10.3390/ijms242417625

**Published:** 2023-12-18

**Authors:** Mariana Pereira, Jéssica Pinto, Belén Arteaga, Ana Guerra, Renato Natal Jorge, Fernando Jorge Monteiro, Christiane Laranjo Salgado

**Affiliations:** 1 i3S—Instituto de Investigação e Inovação em Saúde, Universidade do Porto, 4200-135 Porto, Portugal; mgpereira@i3s.up.pt (M.P.); jessica.p.a.pinto@gmail.com (J.P.); beleniarteaga11@gmail.com (B.A.); fjmont@ineb.up.pt (F.J.M.); 2 INEB—Instituto de Engenharia Biomédica, Universidade do Porto, 4200-135 Porto, Portugal; 3 Faculty of Medicine, University of Granada, Parque Tecnológico de la Salud, Av. de la Investigación 11, 18016 Granada, Spain; 4 INEGI—Instituto de Ciência e Inovação em Engenharia Mecânica e Engenharia Industrial, 4200-465 Porto, Portugal; aguerra@inegi.up.pt (A.G.); rnatal@fe.up.pt (R.N.J.); 5 LAETA—Laboratório Associado de Energia, Transportes e Aeronáutica, Universidade do Porto, 4200-165 Porto, Portugal; 6 FEUP—Faculdade de Engenharia, Departamento de Engenharia Metalúrgica e de Materiais, Universidade do Porto, 4200-165 Porto, Portugal; 7 PCCC—Porto Comprehensive Cancer Center, 4200-072 Porto, Portugal

**Keywords:** angiogenesis, tissue engineering, image analysis, computational biomechanics, biomaterials

## Abstract

One of the complex challenges faced presently by tissue engineering (TE) is the development of vascularized constructs that accurately mimic the extracellular matrix (ECM) of native tissue in which they are inserted to promote vessel growth and, consequently, wound healing and tissue regeneration. TE technique is characterized by several stages, starting from the choice of cell culture and the more appropriate scaffold material that can adequately support and supply them with the necessary biological cues for microvessel development. The next step is to analyze the attained microvasculature, which is reliant on the available labeling and microscopy techniques to visualize the network, as well as metrics employed to characterize it. These are usually attained with the use of software, which has been cited in several works, although no clear standard procedure has been observed to promote the reproduction of the cell response analysis. The present review analyzes not only the various steps previously described in terms of the current standards for evaluation, but also surveys some of the available metrics and software used to quantify networks, along with the detection of analysis limitations and future improvements that could lead to considerable progress for angiogenesis evaluation and application in TE research.

## 1. Introduction

Tissue engineering (TE) is a multidisciplinary field that arises from the necessity to restore, improve, and regenerate damaged tissues caused by various factors such as disease, injury, or congenital disabilities [1,2]. TE strategies allow the evolution of new technologies to develop bioengineered tissues in vitro that mimic specific cellular processes found in vivo. The main components required for TE include the patient’s stem cells, which can differentiate into the desired cell of specific tissue/organ, growth factors that guide cellular behavior, and scaffolds that mimic the extracellular matrix (ECM) [2,3].

The potential to create three-dimensional (3D) scaffold materials that mimic the ECM is currently limited by the capacity to obtain adequately vascularized bioconstructs [4]. Accordingly, in vitro pre-vascularization strategies are essential for TE as pre-formed vascular beds may readily anastomose with the host vasculature and prevent implant failure [5]. Several strategies have been developed to improve vascularization based on two main aspects: scaffold-based and cell-based approaches. In the cell-based approach, angiogenic cells, mainly human umbilical vein endothelial cells (HUVECs), are induced to a state of tube formation when cultured into the scaffolds [6] to create a pre-vascularized construct.

Although the use of endothelial cells (ECs) and the study of their behavior within constructs have become crucial in the field of TE, nevertheless, the parameters for analyzing tube formation, a fundamental component that tends to be an overlooked and underestimated variable, are still not standardized among researchers and image analysis. The proper image analysis techniques allow for a more precise evaluation of the effectiveness of the scaffold in generating the appropriate mechanical and biological stimuli for the development of vascularization in vitro. For instance, if an initial analysis concludes that there is insufficient vessel density from the attained network, culture conditions could be changed, such as by increasing the use of pro-angiogenic factors, or it could even imply a complete change in the design of the bioengineered construct to a more optimized model that would ensure proper vessel maturity [7].

Over the past decades, there have been well-defined goals for the development of angiogenesis imaging analysis that foster the improvement of the cell response quantification and provide rapid assessments; the recently developed software such as REAVER [8] and VesselExpress [9] allow easy imaging analysis execution, reproducibility, and applicability that also could be translated to clinical practice (e.g., cancer treatment). All these data analysis variables still remain relevant, and the continued development of sophisticated imaging techniques has improved data acquisition, although the new high-throughput microscopes provide large data files, which make the adequate interpretation of the studies difficult, potentially compromising the results and the translation into promising clinical therapeutic regimens.

To complement the imaging analysis, the development of mathematical models to describe neoangiogenesis is a rising trend in many clinical areas such as oncology. The models complement the study of in vitro assays and predict, for example, how drugs will impact on neovasculature and therefore on tumor growth. Consequently, computer modeling of neoangiogenesis can be used as an in silico tool to simulate the impact of a variety of biological or synthetic molecules, thus allowing virtual screening and comparisons between different regimens [10].

**Figure 1:**
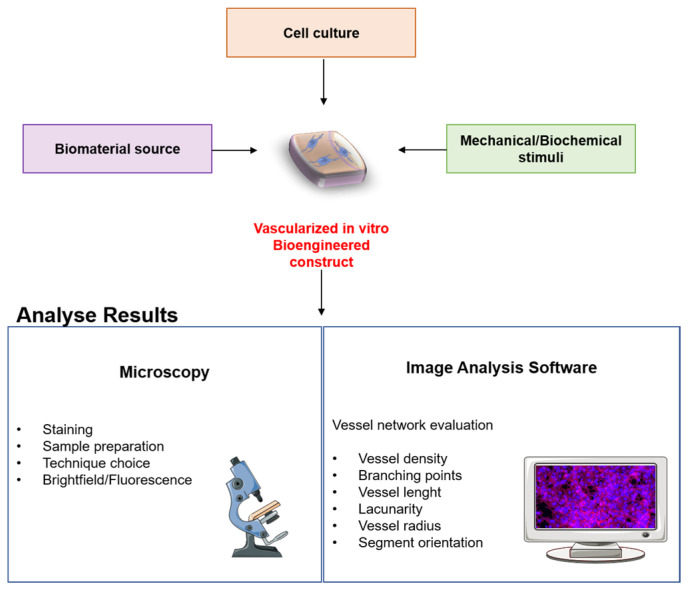
Outline schematic of the themes discussed in the present review. Parts of the figure were drawn by using pictures from Servier Medical Art. Servier Medical Art by Servier is licensed under a Creative Commons Attribution 3.0 Unported License (https://creativecommons.org/licenses/by/3.0/).

A schematic of the themes discussed throughout this review can be found in Figure 1.

## 2. Vascular Tissue

The network of blood vessels connects the heart to all other organs and body tissues. Due to their semi-permeability, the vascular system is in charge of moving fluids, ions, macromolecules, and cells into and out of blood vessels [11].

Blood vessels can be divided into three categories: arteries, veins, and capillaries [12]. Blood capillaries consist of a luminal surface of the monolayer of ECs that form a tube vessel. This thin endothelial tube is supported by the basement membrane around it, which acts to maintain the integrity of this vessel. This layer provides a blood flow channel that is free of friction [13]. This unique design allows for a significant interchange of gases and solutes between the blood and surrounding tissues in the capillary bed, as well as efficient blood flow [13].

### 2.1. Blood Vessel Development

Angiogenesis and vasculogenesis are two similar mechanisms for blood vessel formation. Vasculogenesis occurs with the formation of new blood vessels. Angiogenesis refers to the process of blood vessels formation from an existing one through vascular sprouting [14]. During angiogenesis, quiescent ECs found in an existing vessel are stimulated and activated by the increase in the concentration of pro-angiogenic factors produced by either tumor or inflammatory cells in response to injury and/or hypoxia (reduction in local oxygen concentration) [15].

#### Angiogenesis Mechanism for Adaptation and Stabilization

**Figure 2:**
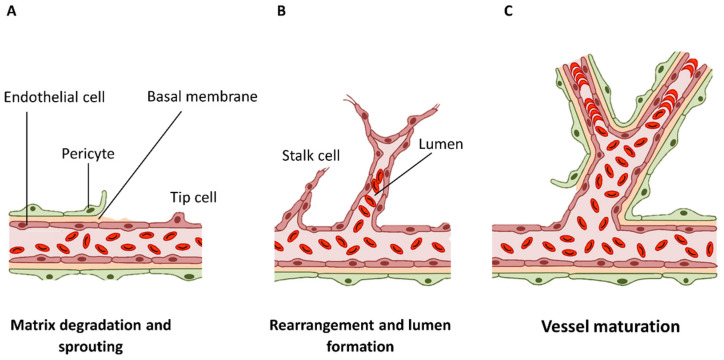
The main stages of angiogenesis are represented schematically: (**A**) breakdown of the basement membrane and vascular sprouting of endothelial cells (ECs); (**B**) appearance of vacuole in ECs and fusion to form a tube-like structure; and (**C**) vessel maturation and stabilization. Cells’ connections are fully repaired (adapted from [16], available via license: CC BY 4.0 DEED).

Angiogenesis can occur in two different forms: sprouting and intussusceptive angiogenesis. In sprouting angiogenesis, new sprouts originate from an already existing vessel and branch to form a new perfused vessel after pro-angiogenic stimulation [13]. Intussusceptive angiogenesis is a dynamic intravascular process capable of dramatically modifying the structure of the microcirculation. Here, a main vessel wall expands and splits, creating two separate vessels from one. The main steps of this process are the degradation of the basal laminae, the endothelial cells (ECs) detachment (matrix and neighboring cells), invasion of the extravascular space (Figure 2A), restructuring of ECs, formation of a lumen (Figure 2B), and, finally, secretion of ECM molecules [16].

Afterward, tube development is followed by vascular regression or vessel stabilization and maturation, meaning ECs undergo apoptosis [17], lose their invasive character, and switch back to a non-proliferative state (Figure 2C) [16].

## 3. Hydrogels Applied in Pre-Vascularization

Tissue regeneration can involve the use of biomaterials as a support surface for cellular growth (e.g., commercial fibrin glue for vascular anastomosis) [18]. These materials that will mimic the extracellular matrix (ECM) need to meet the biomechanical requirements of the target tissue, for example: provide cells a suitable environment, increase cell survival and differentiation, and have an appropriate degradation rate [3].

Hydrogels are a type of scaffold that was first used in the 1960s by Wichterle and Lim for contact lens development [19,20,21,22] but have since been used for a range of biomedical purposes [19,20,21,22]. Hydrogels are one group of scaffolds that is described as polymeric material with 3D hydrophilic polymer chains that are crosslinked by chemical or physical bonding, forming bridges. They could have specific applications such as drug or cell controlled delivery, wound dressing, and biomedical implants [19] due to their porous structure, adequate biocompatibility, and tuneable properties that mimic natural tissues. A hydrogel’s porosity is able to provide a matrix for drug loading and protect molecules from hostile environments at the same time, and the diffusion coefficient can be modified to control the release rate. They also play a crucial role in wound care as they can maintain a moist environment, promoting wound healing [23].

### 3.1. Hydrogel Structure and Properties

A hydrogel’s polymer chains are highly hydrophilic, making them capable of absorbing large quantities of water without dissolving [24]. This is due to the fact that the molecular structure of the hydrogels has significant space between its chains, which allows water uptake of 70–90% of their mass. This depends on the type of monomer that constitutes the hydrophilic chain, the density and type of crosslinking present, and, lastly, external factors such as temperature and pH [25]. However, these biomaterials have poor mechanical strength and stability due to their low viscosity, making their mechanical behavior difficult to test and understand since they behave like neither liquids nor solids [26].

Additionally, mechanical properties can also affect cell behavior, mainly when using 3D materials with different elasticity and viscoelasticity properties. Soft tissues and most hydrogels are viscoelastic [27], meaning that they show both elastic (characterized by stiffness or storage modulus—G′) and viscous (characterized by viscosity or loss modulus—G″) properties. Viscoelastic hydrogels exhibit stress relaxation or creep behaviors under mechanical solicitation. Stress relaxation is observed when stress decreases in response to the same amount of strain applied. These properties can be modulated by controlling their composition, concentration, molecular weight, or crosslinking type/density [28]. The complex modulus (G*) is the sum vector of the viscous and elastic portions. The material’s stiffness increases with the increase in this modulus [27].

The viscoelastic behavior of hydrogels has a direct relation to the different cellular activities. High-concentration gels show a dense network, decreasing cell migration and ingrowth within the 3D matrix of the hydrogel. On the other hand, low-concentration gels show a fluid network structure with lower mechanical properties, and their main problem is that they can easily collapse during implantation. Even with low mechanical strength, injectable hydrogels show an advantage as they can initiate gelation in situ [26].

### 3.2. Hydrogels for Angiogenesis and In Vitro Analysis

Hydrogels can be classified regarding their physical properties, origin, method of preparation, rate of biodegradation, and nature of crosslinked bounding [29,30].

They can be classified into three main categories: natural hydrogels, synthetic hydrogels, or hybrid ones [29]. Synthetic hydrogels have the advantage of being consistent within their mechanical properties, being easy to produce on large scale, which allows the chance to better control and produce constructs that better support the development of angiogenesis [19,31]. Polyethylene glycol (PEG) is one of the more common non-degradable polymers in tissue engineering (TE) as it can be engineered with different materials to increase cellular interaction according to the users’ specific applications [32,33]. Because of this, PEG can be modified to behave as a suitable material for the development of a vascularized construct, either by being coupled with angiogenic factors, such as vascular endothelial growth factor (VEGF) [34], or with natural materials, such as hyaluronic acid [35] and collagen [36]. Another synthetic hydrogel material less studied is poly(vinyl alcohol) (PVA), a hydrophilic and biocompatible polymer that presents a proper setting for the promotion of wound healing due to its solubility and 3D structure [37,38,39]. Despite this, these gels, similarly to PEG and other synthetic materials, are significantly less biocompatible than natural materials and still require modifications to properly resemble the ECM and supply the necessary cues to promote the appropriate [33] cell response, being more typically used to assess how single matrix components may influence the natural development of angiogenesis [19].

On the other hand, natural materials, especially those derived from the ECM, such as fibrin and collagen, show high biocompatibility, low immune response and cytotoxicity, and similar structures to native tissue [22]. Fibrin is considered the most common choice for the study of microvessel development in vitro, usually obtained by mixing fibrinogen with calcium ions and thrombin [40,41]. This material not only possesses intrinsic angiogenic abilities, but, as it is involved in the process of wound healing, also supports the invasion of endothelial cells, facilitating the neovascularization of wound sites [33]. The fibrinogen can also be sourced directly from human plasma, ensuring higher cell viability for the implanted hydrogels [32,42]. It should be pointed out, however, that fibrin constructs tend to degrade fast in vivo and show poor mechanical properties, limiting the use of this material by itself [43]. Similarly, collagen, especially type I, is also presented as a suitable hydrogel material for the study of angiogenesis in vitro as it is the more prevalent protein in the ECM of the connective tissue [43,44]. However, collagen’s degradation products are considered to have a thrombogenic effect and initiate the coagulation cascade [43,45], limiting the use of this material by itself; its properties could be improved by crosslinking or mixing with other materials, such as elastin [45]. Due to the characteristics of natural scaffolds, they have a great potential for various TE applications, especially for the development and analysis of angiogenic models [19].

## 4. Imaging and Analyzing Assays in Angiogenesis

With the development of newer in vitro models for microvessel networks, it becomes imperative to properly collect all the necessary data from these experiments to gain insights into the molecular, cellular, and functional aspects of angiogenesis. For this, researchers often rely on imaging and analysis techniques, which serve a multifaceted purpose, as they not only enable the user to identify cellular structure and morphology, but also evaluate factors like the location, quantity of new blood vessels, morphological characteristics, and permeability of blood vessels. Additionally, imaging analysis helps in the identification of vascular markers and assessing the impact of compounds on angiogenesis. The crux of these imaging techniques lies in the ability to quantify parameters like endothelial migration, proliferation, sprouting, or the overall tubular network [46].

### Microscopy Techniques: Optical and Fluorescence Microscopy

Transmitted or brightfield light optical microscopy is one of the most common methods of imaging for biological specimens. The core principle of this technique is the transmission of light through a condenser lens onto the specimen, which allows fast image acquisition. Since different biological components within the specimen absorb varying amounts of light, this method generates image contrast which allows visualization of different components within the specimen. Phase-contrast microscopy uses a similar imaging system where the image is dependent on the amount of light obtained from a biological specimen. However, thin biological specimens such as ECs absorb only small amounts of light, leading to low-contrast images with unclear object boundaries. For accurate analysis, clear distinctions between the object of interest, background, and other biological components are required. To address this, cells or proteins within the specimen can be labeled or stained to enhance the signal [47]. Overall, sample preparation for this technique is quite simple as these materials are simply placed under an inverted microscope to observe vessel development and be later analyzed via image analysis software [48,49]. Therefore, to select samples to have their vascular network analyzed in more detail, it could be performed using more complex imaging strategies, such as dyes that can be used in sample preparation to better contrast the cells (such as crystal violet [50], safranin [51], and methylene blue [52]); these do not allow for a more in-depth observation of cellular organelles [53,54].

Fluorescence microscopy, another powerful optical technique, shares similarities with brightfield microscopy in terms of image acquisition but uses specific wavelengths of light to excite fluorophores within the specimen. Fluorophores are chemical compounds that emit light when returning to their original energy state, and this emitted light is differentiated from the excitation light by spectral emission filters and dichroic mirrors. By choosing a specific wavelength to excite these fluorophores, researchers induce the emission of light. For these samples, the materials are first fixed with either formaldehyde, paraformaldehyde, or formalin. A main concern when using formalin (100%) is the presence of methanol (stabilizer), which results in permeabilization of the membrane and an interference in the staining of bound proteins on it. After that, the cells are permeabilized with mild, non-ionic surfactant (e.g., Triton X-100), which will allow the access of the antibodies (immunostaining) to the cell cytoplasm without membrane disruption. Finally, before using antibodies staining, the sample is incubated with a protein solution to “block” the sample to reduce non-specific binding of the selected fluorescent antibodies [55]. For specimens with multiple fluorophores, images for each fluorophore must be acquired in different channels and finally combined [56].

The advantage of fluorescence microscopy lies in its applicability to both fixed and live biological specimens, which offers a higher signal-to-background ratio in comparison to brightfield microscopy [57]. Confocal fluorescence microscopy, such as confocal laser scanning microscopy (CLSM), can offer significant advantages as it excludes out-of-focus light, resulting in higher image resolution; only light emitted from fluorophores from within the focal plane of the specimen is detected in confocal microscopy [58].

Optically sectioning the specimen along the *z*-axis using confocal microscopy further enhances resolution by capturing images from different focal planes. Fluorescence microscopy has proven invaluable for studying the molecular and functional aspects of angiogenesis, enabling dynamic tracking of proteins, identification of interactions, and non-invasive whole-body imaging. However, it has limitations, including photobleaching, spectral cross-talk, and specimen phototoxicity, which can affect image quality and cellular viability [59]. Along with this, after the image is acquired, the next step is to analyze the formation of the vascular network, that is, the number of vessels, segments, branches, and junctions formed throughout the studied material. For this, several pieces of image analysis software have been developed throughout the years with the express intent of automatically performing this type of analysis.

Therefore, there are other available state-of-the-art techniques that are mostly non-optical imaging methods such as X-ray, computed tomography (CT), and magnetic resonance imaging (MRI). These are used to evaluate the integration of tissue engineering (TE) constructs and their structural changes in vivo or ex vivo since in vitro samples have low density and contrast and allow the use of techniques other than optical light, fluorescent, or two-photon microscopy. Some other non-optical methods are often used in clinical settings for vascular evaluations in vivo, including ultrasound and positron emission topography (PET). These techniques have excellent tissue penetration and coverage (1 cm–1 m) but with lower spatial resolution (0.1–10 mm) [60], which is more suitable for the evaluation of relatively larger-diameter vessels (0.1–1 mm) in a patient [61]. Preclinical imaging of TE constructs on animal implants requires much higher spatial resolution because the blood vessels in small animals (e.g., mice) are much smaller than in humans [7]. On the other hand, sonography allows for the visualization of vascular architecture and flow rates in several organs, although contrasting agents are recommended to overcome the technique’s poor specificity [62].

## 5. Image Analysis

**Figure 3:**
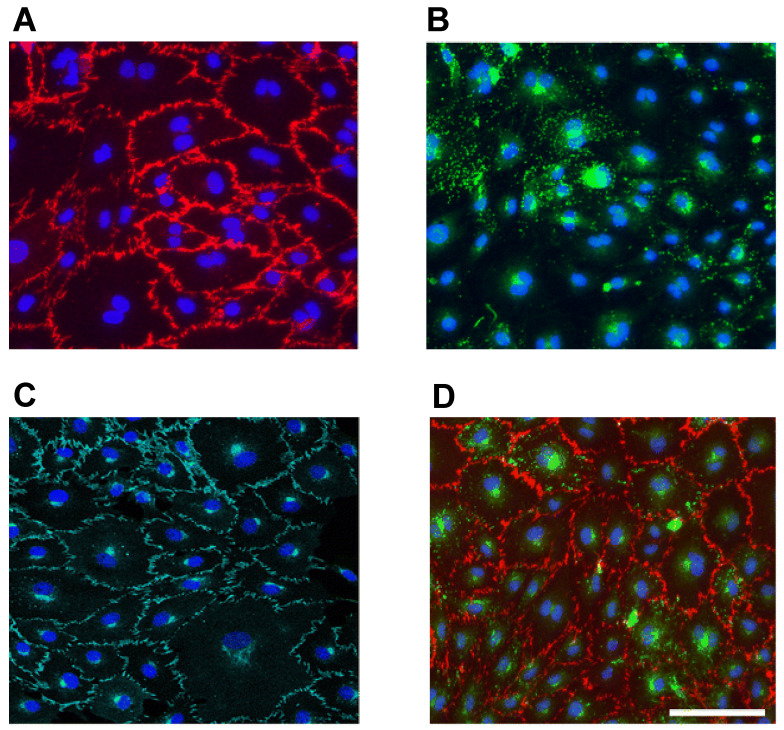
Example of ECs successfully detected via (**A**) CD31 membrane staining and (**B**) von Willebrand factor (vWf) cytosol staining, (**C**) VE-cadherin membrane staining and (**D**) CD31 membrane staining and vWf cytosol staining. Scale bar = 100 µm (adapted from Ref. [70], available via license: CC BY 4.0, DEED).

To properly evaluate the newly formed microvascular network, a suitable labeling that constitutes it is essential for attaining an image of the contrasting microvessels against the encompassing construct, with the choice of the particular labeling marker to be used being determined by the objective of the research itself, such as a focus on angiogenesis development or characterizing blood flow [63]. Specific cell markers can be identified in either the cytoplasm or the membrane of endothelial cells (ECs), with some being expressed for all types of ECs and others remaining limited to certain tissues only. Some of the classical antigens to evaluate endothelial cell morphology are CD31 (Figure 3A) and VE-cadherin (Figure 3B). CD31 is a glycoprotein found in the membrane of ECs, being considered their universal biomarker [64,65,66] and identified via immunofluorescence staining [67,68]. Similarly, VE-cadherin is an adhesion molecule that is explicitly found in endothelial junctions [69], playing an important role in the maintenance of vascular integrity [64,67]. Because of this, the expression of this marker in a scaffold, via immunofluorescence analysis, indicates a proper interplay between the construct and the ECs [68].

**Figure 4:**
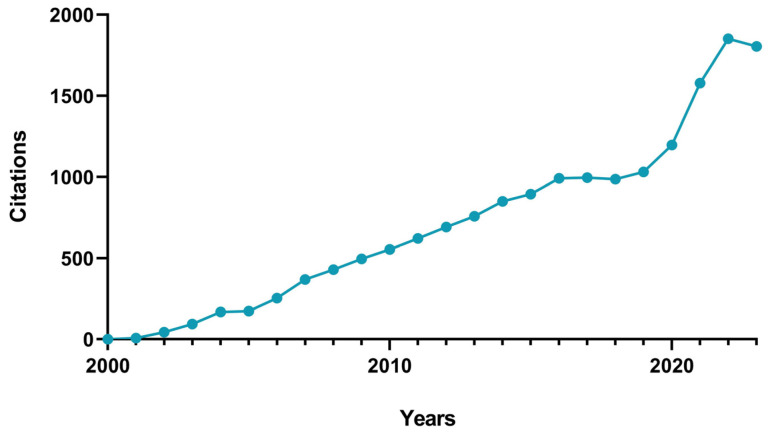
Citations per year chart from the range of 2000–2023 in the Scopus^®^ database referent to the keywords “angiogenesis AND image AND analysis AND in AND vitro”.

With the cells properly labeled, the image acquisition follows, and, later on, the attained images are run through suitable image analysis software to quantify the different metrics that characterize the microvasculature network. These metrics include, but are not limited to, vessel length density [71,72,73], average vessel diameter [72], branchpoint density [74], and fractal dimension [75,76]. Due to the complexity in properly and correctly identifying these unique characteristics, it becomes crucial to select appropriate image analysis software, as well as the parameters to be used, in accordance with the purpose of the study. There has been considerable interest in the imaging analysis of in vitro anagenesis assays over the years, with a Scopus search (using the keywords “angiogenesis AND image AND analysis AND in AND vitro”) in the interval of 2000–2023 yielding 519 document results, including articles (458), reviews (28), conference papers (16), book chapters (10), errata (4), conference reviews (1), editorials (1), and notes (1). From these, a citation chart was attained (Figure 4), and a notable increase in the published articles was observed over the decades, especially between 2021 and 2023.

### 5.1. ImageJ

ImageJ software (most recent version 1.54g, as of October 2023) was developed by Wayne Rasband in 1997 as a successor to NIH Image [77,78], consisting of an open-access image-processing program with a built-in editor and Java compiler which allow users to freely personalize the program’s code with custom-built processing, analysis, and acquisition plugins to solve various image processing issues [48,79]. These characteristics, along with its straightforward use and the fact that it can run on any operating system, make ImageJ one of the more commonly used pieces of image analysis and processing software used for the study of angiogenesis development [78].

Several papers on angiogenesis analysis mention using ImageJ as the main, if not the only, image analysis software for assessing the development of the normal progression of angiogenesis. Various authors have accomplished this by using ImageJ to access several parameters; however, most of these studies do not expand upon the steps and parameters applied in ImageJ to attain their results, or even mention whether changes had to be made to its code, with many only mentioning the use of the software itself, making reproducibility of the image analysis process in angiogenic studies a challenging step for other users [53,72,73,80,81,82,83,84,85,86,87,88,89,90,91,92,93,94,95,96,97,98,99,100,101].

On the other hand, the few studies that do provide detailed information on their use of ImageJ demonstrate the program’s versatility for the study of angiogenesis development as several authors were able to show not only changes in its code, but also the use of various plugins to better adapt the program to a specific study. The input of these types of plugins and macros is made easier by the use of Fiji, a distribution of ImageJ that aims to provide a carefully organized selection of various plugins and libraries [79].

**Figure 5:**
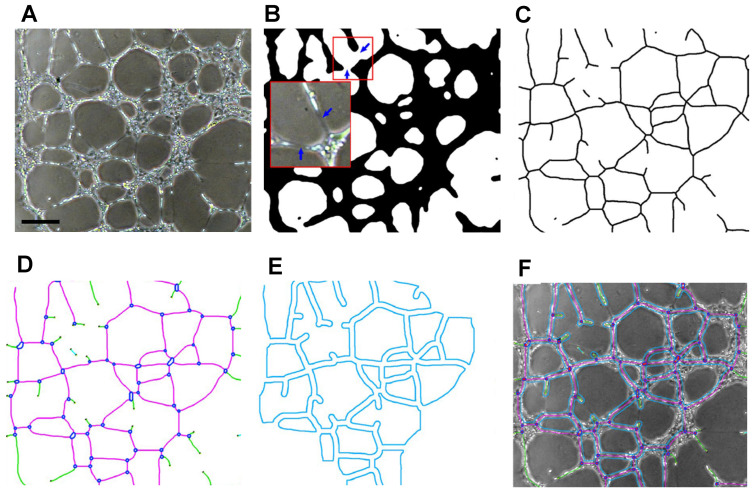
Example of HUVEC network analysis in ETFA (adapted from Ref. [102], available via license: CC BY 4.0 DEED), Scale bar: 75 µm. (**A**) Amplification of the HUVEC network, followed by attaining a (**B**) binary image (blue arrows represent how the “mask” was smoothed from the original image) and (**C**) a skeleton of the binary segmentation, from which (**D**) detection of the segments (pink), branches (green), junction (blue), and extremities (dark dots) is performed, and, finally, an (**E**) outline of the meshes is attained. (**F**) Initial image of the vessel with an overlap of the vectorized objects.

One of the more cited plugins used in ImageJ is the “Angiogenesis Analyzer”, developed by Carpentier in 2012. This program was initially created as a way to better extract and quantify characteristic points of a vessel network in Endothelial Tube Formation Assays (ETFA) [102] and has been continuously updated throughout the years, being considered to be an efficient and easily reproducible method to detect many angiogenic parameters, such as tube length, number of branches, and number of junctions, in both 2D and 3D [74,103,104,105,106]. A detailed explanation of the use and workings of the program is provided by Carpentier, but, simply put, the Angiogenesis Analyzer starts by creating a binary mask (Figure 5A), which is then segmented (Figure 5B), creating a skeleton (Figure 5C), or tree, from which extremities, nodes, and junctions are first detected via the image’s pixels (Figure 5D). From these, segments and branches are identified from the various bifurcations noticed in the skeleton, and a final analysis is made, in which the main structure of the network is identified by detecting the meshes (Figure 5E), considered to be areas encompassed by segments and junctions, and removing more artificial branches. An Excel file is returned at the end, with many parameters that were analyzed, with similar results having been attained with both phase-contrast and fluorescence models [102,107]. The Angiogenesis Analyzer has also been used for other angiogenic models, such as the Fibrin Bead Assay (FBA) [108,109]. More recently, a new algorithm was added to the Angiogenesis Analyzer which performs an automated analysis of this FBA assay with phase-contrast microscopy [102]. This performs the same steps as mentioned for the ETFA, with the added first step of sphere detection. However, several factors, such as bead clustering, the presence of dust, and uneven lighting, may affect the accuracy of this analysis, which involves quite a bit of image manipulation. Despite this, this algorithm has been shown to effectively return a meticulous model of the vascular network and the beads, as well as quantify several parameters related to neovessel formation, including the number of capillaries formed from the beads, extremities, and branches and network length, all of which can be performed with no user intervention [102,110].

Eglinger et al. (2017) [81] developed another plugin for the Fiji distribution of ImageJ which is used for a bead sprouting assay and automatically quantifies sprout morphology of HUVECs and pericytes microbeads inserted in a fibrin gel. This bead sprouting assay, which allows the accurate measurement of beads, vascular sprouts, cell density, and area covered with pericytes, is described step by step in the published work. The code for the plugin is also available online and used by other authors [81,111]. Kempers et al. (2021) later expanded on this plugin by creating a macro, “Automated sprout analysis”, for fast automated quantitative analysis of sprouting of HUVECs in fibrin gels [112]. This macro saves the raw confocal images, enhances the contrast, and stores the obtained results in separate folder names, along with an Excel file with the used parameters and results, namely, the number of beads, nuclei, and sprouts and network length. However, it is mentioned that large amounts of RAM are required (at least 16 GB) for both the macro and ImageJ to function properly; otherwise, the program will halt the analysis. Additionally, despite its user-friendly front, ImageJ itself requires some level of insight related to computer programming, making it not always the very first choice of various researchers [113]. Because of this, ImageJ may be placed in the side-lines of various angiogenic studies, or, at least, be not fully used by itself.

### 5.2. Alternatives to ImageJ

As mentioned, although ImageJ is still the most cited software for performing image analysis in several angiogenic studies, several researchers have chosen to not use it as the main program.

**Figure 6:**
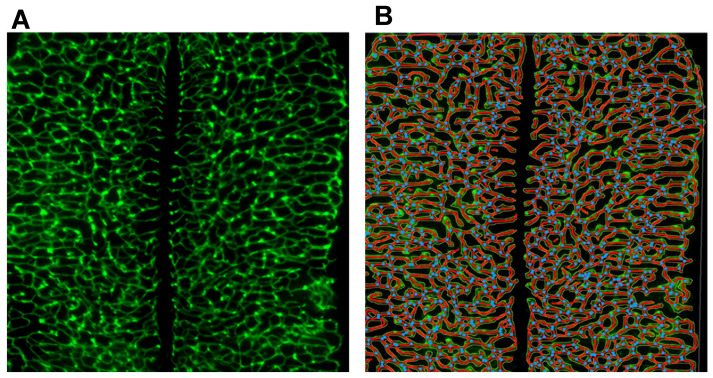
Example of a microvessel image analysis performed by AngioTool (adapted from [71], licensed by CC 1.0 DEED). (**A**) Original vessel network attained from an embryonic hindbrain culture and (**B**) final image of the analyzed network with the represented outline (yellow), skeleton (red), and branching points (blue).

A well-known piece of software for angiogenesis analysis is AngioTool (software version 0.5, as of 2011), developed by Zudaire et al. (2011) as a response to the lack of a standardized, user-friendly software and automated software in quantitative analysis of the vascular networks of embryonic hindbrain, the post-natal retina, and allantois explants [71]. This program firstly identifies the vessels in the provided image according to pre-setting parameters, including, but not limited to, vessel density, number of junctions, branching index, and average vessel length. Therefore, the vessels are delimited with an outline which changes in accordance with adjustments made by the user (segmentation and skeletonization). The attained “skeleton” of the vessel network is analyzed, resulting in an image with all the identified vessels and branching points (Figure 6B), as well as an Excel file containing the analyzed parameters and results. Some strong points of this plugin are noted, such as the ability to adjust the software’s pre-setting parameters to better detect fine vessels and the automation of tasks that would otherwise be performed by the human eye, namely, counting the number of junctions in a vascular network image, thus reducing the probability of human error.

However, some referenced shortcomings include the fact that the compatibility of the results is dependent on the very first step of the plugin, that is, the optimization of the skeletal network, which is dependent on the software’s pre-setting parameters. For example, the software allows the changing of these parameters to better detect finer vessels, such as choosing many vessel diameter scales and intensity settings, because of which there is a noted risk of the program wrongfully identifying vessels. Despite this, AngioTool has been used to successfully assess the formation of a vascularized network in various other scenarios, namely, vessel sprouting from spheroids [114], a 3D-printed resin [115,116], poly(L-lactide)/poly lactic-co-glycolic acid (PLLA/PLGA) scaffolds [117], and fibrin hydrogels [118], making it a more reliable and reproducible image analysis device [63].

Therefore, some pieces of software that can be used by themselves for angiogenic image analysis are mostly found being used together with ImageJ, such as Amira and WinFiber3D.

Firstly, Amira software was initially developed by the Zuse Institute Berlin (ZIB) and is currently commercially available from Thermo Fisher Scientific. This program allows the visualization, analysis, and processing of 3D biological images [119]. Some of the more attractive features of this program consist of image segmentation, as well as geometry reconstruction, in which different bodies of interest presented in the image can be identified and enhanced, and, later on, this model can be smoothed so as to not lose small details from the original dataset [119]. Other features, such as skeletonization [120], 3D reconstruction, deconvolution, binarization [121], and noise removal [122], have made Amira quite an attractive piece of software for image analysis of biological datasets. However, it is important to note that, unlike ImageJ and AngioTool, Amira is not freely available software, which may limit its use.

On the other hand, WinFiber 3D is a piece of custom software initially created to quantify vascular networks and allows the visualization of MicroVisu3D files (.mv3d). Because of this, the software is not typically observed used by itself, but usually together with other software, such as Amira [123]. The program can successfully quantify other types of 3D networks, as well allowing the analysis of network distribution statistics, such as segment orientation, length, vessel length within a defined range, and branchpoints [123,124].

Because of this, WinFiber3D has been observed as a useful aid to other programs, such as ImageJ/Fiji and Amira, in the analysis of vessel development. Examples of this can be observed and are described in detail in several works [121,122,125]. Boyd et al. (2013) have described their use of various software, along with schemes that better explain this process. Firstly, ImageJ was used to convert confocal stacks of 3D confocal images of HUVECs transduced with DsRed into 8-bit grayscale and Amira to correct image depth and deconvolve and binarize the attained pictures [121]. On the other hand, 2D fluorescence images were converted to grayscale, binarized, and filtered by size using MATLAB, a piece of software that has also been observed to be an aid for network image analysis [126,127]. Amira software was then used to perform skeletonization of the vessel network in the binarized images, from which the number of vessels and branchpoints segments, as well as vessel lengths and diameters, were attained in WinFiber3D.

On the other hand, Davern (2020) firstly corrected confocal z-stacks of HUVECs stained with anti-CD31 primary antibody, Streptavidin Alexa Fluor 488 and 4′-6-diamidino-2-phenylindole (DAPI), to attain more homogenous fluorescence levels [122]. ImageJ was then used to convert these into “multipagetiff” files, and to convert images into monochrome, with intensity signals separated into black and white groups after these had undergone a noise reduction process using Amira. Finally, similarly to what was previously described, Amira was used to identify vessel structures (skeletonization), which were quantified in WinFiber3D.

Finally, other software worth mentioning in this review include those such as AngioQuant, RAVE, REAVER, and VesselExpress.

AngioQuant (software version v1.33, 2005), a MATLAB-based piece of software, was initially developed for the automatic quantification of in vitro angiogenesis of endothelial cells (ECs) co-cultured with fibroblasts as these types of cultures are typically used for the study of anti-carcinogenic pharmaceuticals. Specifically, this software was developed to provide a more accurate quantification of the development/inhibition of a complex vessel network to evaluate cancer treatment via the quantification of tubule complexes [49,128]. This process is described in detail by Niemisto et al. (2005), but, similarly to other previously mentioned software, AngioQuant first performs a binarization of the network image to enhance the connected tubules, which are darker than the foreground and the fibroblasts, from which the tubules are skeletonized and several parameters are measured, such as total and average vessel length and size and number of junctions and branching points. A drawback that is pointed out is that, preferably, this image should be attained as soon as the angiogenesis experiment is finished to ensure a strong enough staining and contrast [49]. Additionally, the software has been updated to analyze in vivo samples, such as the CAM assay [128,129,130], although its performance is not fully agreed between users, with some authors considering the software to have a long running time [131].

Later, a MATLAB-based piece of software), referred to as RAVE (Rapid Analysis of Vessel Elements, software version v1.2, 2011), was developed by Seaman et al. (2011), who stated that the software has a more accurate and rapid (seconds to minutes) analysis for different vessel parameters [75], such as vessel volume fraction, vessel length density, fractal dimension, and mean vessel radius, of in vivo samples from mice muscle tissue and a xenograph tumor model, along with other samples, when compared to manual analysis [75,132,133]. Furthermore, RAVE was capable of distinguishing regular pancreatic vasculature from tumor-associated vessels via a significant change in the previously mentioned parameters. Despite this, the authors mention that a drawback is that their software cannot be easily modified to perform a three-dimensional (3D) vessel analysis and cannot analyze flow and branch angle. Consequently, RAVE cannot ascertain a vessel’s tendency for movement in a z-plane, although a suggestion is made to perform it in two dimensions [75].

More recently, Rapid Editable Analysis of Vessel Elements Routine (REAVER, 2023), an open-source tool that researchers can use to analyze high-resolution 2D fluorescent images of blood vessel networks has been developed. When manually analyzed, REAVER exhibited high accuracy and precision for all vessel architecture metrics quantified on tissue slices, (e.g., vessel length density, branchpoint count). Although the automated segmentation is inaccurate, the authors show that combining manual curation with automated analysis improves the accuracy of vessel architecture metrics [8]. Finally, this year, an open-source and platform-independent piece of software, VesselExpress (software v1.1.1, 2023), was launched to fully automatically analyze light-sheet fluorescence microscopy (LSFM) 3D data of blood vessel systems. It allows fast image analysis, processing, and graph construction. It also enables high-volume analyses, able to extract the microvascular network (length, branching, and diameter). But, labeling with endothelial-specific antibodies results in hollow tubes that are not optimized yet since the software was optimized with fluorescent hydrogel injected into the vasculature [9].

A summary of the described image analysis software, along with advantages and drawbacks, is outlined in Table 1.

### 5.3. Future Perspectives

Despite all the presently available image analysis tools, a stark difference has been noticed between the number of publications that mention various metrics related to the quantification and development of vessel architecture and the number of citations referring to the image analysis software itself [63]. This noticeable disparity is proposed to be due to shortcomings noticed with most commercially available tools [71,77,79,81,107] that severely limit the correct analysis of the architecture of the microvessel structure [111].

Most of these programs perform the quantification of branch density, area, length, and migration of the vessel’s thresholding-based segmentation, which, especially if the attained vascular architecture is considerably complex, may severely compromise the exactness of the final segmentation [136]. Since most image analysis tools do not quantify the vacant area within the lumen, the results critically limit the data related to the vessel’s development and functionality [111]. Because of this, researchers end up relying more on manually quantifying the various parameters of the microvessel networks, considerably reducing the reproducibility and accuracy of the analysis [63].

With this, novel approaches have been developed for the automated quantification of angiogenic sprouting for different assays, such as the automated segmentation of the endothelial lumen space [111]. More noticeably, new image analysis tools based on the use of deep learning and artificial intelligence (AI) algorithms have emerged as a new solution to accurately measure different metrics of blood vessels from various tissue models, such as the retinal microvasculature [137,138,139], chorioallantoic membrane (CAM) model [140], and, more commonly, tumor models [141,142,143]. Ramakrishnan et al. (2023) introduced deep-learning-based algorithms that could reconstruct the 3D model of blood vessels from parallel tissue sections, independent of the applied staining technique, which could present numerous research applications in the future, such as to rebuild tumor vessels, and better understand the vasculature development in these in vivo models [144]. However, the use of these types of algorithms also presents shortcomings. Firstly, the appearance of the blood vessels is dependent on the preparation of the staining and the slides, which will affect the analysis performed by the AI algorithm [145]. Additionally, these in vivo models can be considered to be difficult to interpret [146], compromising their more widespread use in a clinical setting and workflow, along with raising concerns about data protection and misdiagnosis [145].

Even with newer developments continuously appearing, it has been discussed that there are still considerable measures which should be taken in order to improve already commercially available programs. Corliss et al. (2018) have previously enumerated several guidelines that could be followed for the development of a more efficient vessel image analysis software [63]. Some of these include the assessment of novel metrics for the characterization of the microvascular network in order to attain more information for the advancement of more complex in silico models that more closely resemble the experimental models [147,148], which can be compromised if the software itself does not warn of any limitations within the provided image. This insufficient data decrease the accuracy of the different vascular metrics and may also need improvement by providing biological datasets with the imaging of microvascular systems from different types of tissues, ensuring that the software can accurately identify and distinguish valid vessel metrics [63]. Similarly, unanimity must be achieved in terms of how these same metrics are measured, as each piece of software tends to measure the same metric using different methods, which may result in disparity in results for the same assays. Finally, current image analysis tools tend to perform the segmentation process over 2D projections of the original 3D structures, resulting in the possible alteration and even loss of the original metrics, as well as not fully providing information on the position of the different vessels in the construct, with overlapping branches in a 2D projection originally corresponding to different points in the 3D construct [63,111].

Finally, although the image analysis software, especially for the analysis of angiogenesis development, has come a long way since its initial development, several improvements can still be made in order to assure more reliability and reproducibility in microvascular analysis; otherwise, research into this field may be affected by false negatives and unreliable results.

## 6. Concluding Remarks

As noted throughout this review, several guidelines are in place in the development of a microvascular network, from more appropriate cell culture protocols to different types of materials to be used (synthetic, natural, or a mixture of both) and even which type of labelling is more suitable in accordance with the studied tissue and the expected biological outcomes. However, similar outputs cannot be taken from vascular image analysis. Although several papers have mentioned the use of different image analysis software for this purpose, from most widely known to rather used, it has been shown to be considerably challenging to find citations that either describe in detail (even in supplementary data) the steps that were taken to perform the image analysis for vasculature development, or which parameters were selected/changed to attain the final analysis. This lack of reporting by researchers diminishes the reproducibility and discussion of the various endothelial sprouting assays and compromises the development of a proper standard from which all these experiments could start. In addition to the suggested improvements for the current image analysis software needs, we also suggest better reporting by the users in terms of steps taken throughout their use of the available image analysis tools as the means to further advance the microvascular development research.

## Figures and Tables

**Table 1:** Summary of different angiogenesis image analysis software.

Software	Input	Output	Advantages	Disadvantages	References
ImageJ (“Angiogenenis Analyser” and “Automated Sprout Analysis”)	Phase-contrast or fluorescence 2D images	Area covered by the cells, total network length, number of meshes, nodes, extremities, and isolated elements, length of segments and branching interval	Runs on any operating system; Code can be customized according to the users’ objectives; Automatic process	Requires large amounts of RAM; Some level of computer programming may be required, depending on the objective; Mostly dependent on custom plugins and macros. Some plugins may involve image manipulation to fully function	[48,78,79,102,113,134]
AngioTool	2D fluorescence images	Explant area, vessel density, branching index, number of endpoints, lacunarity, and total and average vessel length	Parameters can be adjusted to better define the vessels; Automatic process with a lower chance of human error	Results are dependent on how the initial parameters are set; Wrongful detection of vessels	[71]
Amira	2D and 3D images	Vessel volume fraction, vessel length density, fractal dimension, and mean vessel radius	3D reconstruction, noise removal, quantification of 3D networks	Not freely available	[119,120,121]
WinFiber3D	3D fluorescence images	Segment orientation, average and total vessel length, even in a defined range, number of vessels and diameter	Can analyze segment orientation	-	[121,122,124,135]
AngioQuant	2D brightfield images	Vessel length, segment area, branchpoints, segment count	Designed for co-culture assays; Can be used for CAM assays	Long running time	[63,131]
RAVE	2D fluorescent images	Vessel volume fraction, vessel length density, fractal dimension, mean vessel radius	Rapid analysis; Detects differences between healthy and tumor-associated vasculature	Cannot be modified to perform 3D vessel analysis	[75,132]
REAVER	2D fluorescent images	Vessel length density, vessel area fraction, branchpoint count, mean vessel diameter	High pixel-by-pixel accuracy for vessel segmentation	Automated segmentation is considered inaccurate, and it is recommended to combine it with manual assistance	[8]
VesselExpress	LSFM 3D data of blood vessels	Microvascular length, branching, diameter, tortuosity	Fast image analysis, processing, and graph composition; High-volume analysis	Hollow tubes show up if endothelial-specific antibodies are used	[9]

## Data Availability

No new data were created or analyzed in this study. Data sharing is not applicable to this article.
